# Comparison of mouse models of microbial experience reveals differences in microbial diversity and response to vaccination

**DOI:** 10.1128/msphere.00654-23

**Published:** 2024-01-29

**Authors:** Autumn E. Sanders, Henriette Arnesen, Frances K. Shepherd, Dira S. Putri, Jessica K. Fiege, Mark J. Pierson, Shanley N. Roach, Harald Carlsen, David Masopust, Preben Boysen, Ryan A. Langlois

**Affiliations:** 1 Department of Microbiology and Immunology, University of Minnesota, Minneapolis, Minnesota, USA; 2 Faculty of Veterinary Medicine, Norwegian University of Life Sciences, Ås, Norway; 3 Center for Immunology, University of Minnesota, Minneapolis, Minnesota, USA; 4 Faculty of Chemistry, Biotechnology and Food Science, Norwegian University of Life Sciences, Ås, Norway; Emory University School of Medicine, Atlanta, Georgia, USA

**Keywords:** mouse models, vaccines, natural mouse viruses, preclinical models

## Abstract

Specific pathogen-free (SPF) laboratory mice dominate preclinical studies for immunology and vaccinology. Unfortunately, SPF mice often fail to accurately model human responses to vaccination and other immunological perturbations. Several groups have taken different approaches to introduce additional microbial experience to SPF mice to better model human immune experience. How these different models compare is unknown. Here, we directly compare three models: housing SPF mice in a microbe-rich barn-like environment (feralizing), adding wild-caught mice to the barn-like environment (fer-cohoused), or cohousing SPF mice with pet store mice in a barrier facility (pet-cohoused); the two latter representing different murine sources of microbial transmission. Pet-cohousing mice resulted in the greatest microbial exposure. Feralizing alone did not result in the transmission of any pathogens tested, while fer-cohousing resulted in the transmission of several picornaviruses. Murine astrovirus 2, the most common pathogen from pet store mice, was absent from the other two model systems. Previously, we had shown that pet-cohousing reduced the antibody response to vaccination compared with SPF mice. This was not recapitulated in either the feralized or fer-cohoused mice. These data indicate that not all dirty mouse models are equivalent in either microbial experience or immune responses to vaccination. These disparities suggest that more cross model comparisons are needed but also represent opportunities to uncover microbe combination-specific phenotypes and develop more refined experimental models. Given the breadth of microbes encountered by humans across the globe, multiple model systems may be needed to accurately recapitulate heterogenous human immune responses.

**IMPORTANCE:**

Animal models are an essential tool for evaluating clinical interventions. Unfortunately, they can often fail to accurately predict outcomes when translated into humans. This failure is due in part to a lack of natural infections experienced by most laboratory animals. To improve the mouse model, we and others have exposed laboratory mice to microbes they would experience in the wild. Although these models have been growing in popularity, these different models have not been specifically compared. Here, we directly compare how three different models of microbial experience impact the immune response to influenza vaccination. We find that these models are not the same and that the degree of microbial exposure affects the magnitude of the response to vaccination. These results provide an opportunity for the field to continue comparing and contrasting these systems to determine which models best recapitulate different aspects of the human condition.

## OBSERVATION

Humans experience complex infections from eukaryotic, prokaryotic, and viral microorganisms throughout their lives. These include acute resolving infections, chronic infections, and commensal microflora. These experiences shape the immune system at baseline and impact the response to new infections. Researchers have removed most pathogen exposures from laboratory mice to reduce confounding experimental variables. However, these specific pathogen-free (SPF) animals have less activated and experienced immune systems (1–6) with negative impacts on the translatability of preclinical studies (7–9).

We and others have previously shown that SPF mice do not recapitulate human immune responses to vaccination and that introducing microbial experiences yields vaccine responses that better recapitulate those observed in humans (6, 7). There are several models that have been developed to reintroduce natural mouse and environmental pathogens to laboratory mice to better recapitulate the human immune system (10). However, no one model can capture the complexity of human exposures across space and time. Additionally, there have not been any cross comparisons between these models, which has remained a major gap in the field. Here, we directly compare the vaccine responses in three different models of microbial experience introduction. Using the same vaccine regimen, we found that only the model with the largest diversity of microbes had an impact on the magnitude of the vaccine responses. These data suggest that, similar to what is experienced by humans, the heterogeneity in natural microbial experience can impact *de novo* immune responses and further highlight the need to cross compare models of microbial experience.

## RESULTS

### Microbial experience varies across different model systems

**Fig 1:**
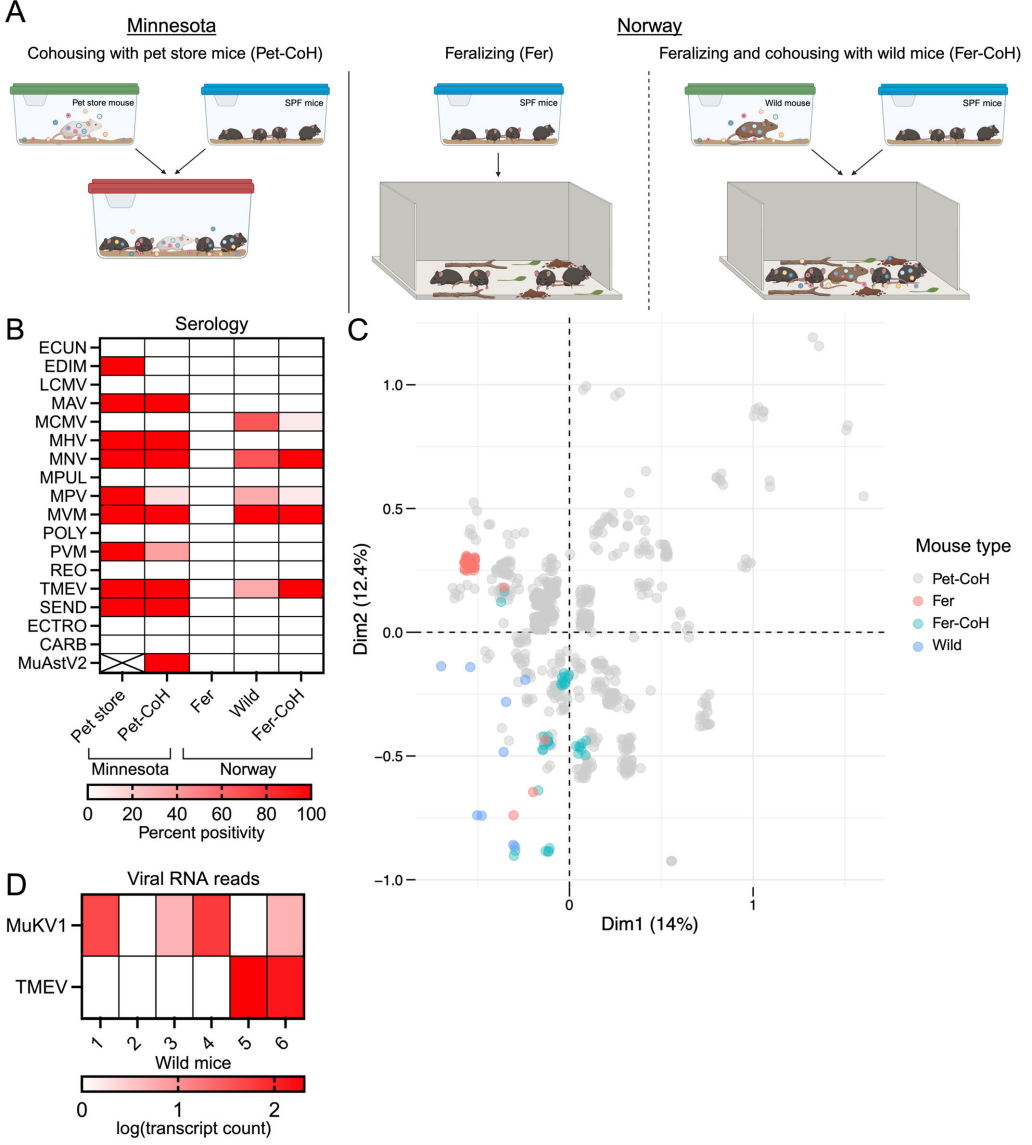
Comparison of three model systems of microbial exposure. (A) Diagrams of the model systems: cohousing SPF mice with pet store mice (pet-cohoused, Pet-CoH), feralizing SPF mice in pens (feralized, Fer), or feralizing SPF mice in pens and cohousing them with wild mice (feralized-cohoused, Fer-CoH). (B) Heat map of serology from pet store, pet-cohoused, feralized, wild, and feralized-cohoused mice at 78–84 days post-cohousing. Pet store *n* = 2, pet-cohoused *n* = 8, feralized *n* = 5, wild *n* = 3, and feralized-cohoused *n* = 12. (C) Multiple correspondence analysis biplot of serology from pet-cohoused mice from Minnesota and feralized, wild, and feralized-cohoused mice from Norway (pet-cohoused *n* = 656, feralized *n* = 32, wild *n* = 9, and feralized-cohoused *n* = 36). Distances represent similarities between pathogen exposure as determined by serostatus. (D) Wild-caught mice in Norway were sampled for viruses by RNAseq of spleen, liver, lung, and intestines. All viruses with reads greater than 1 read/million are shown. Wild *n* = 6. TMEV, Theiler’s murine encephalomyelitis virus and MuKV1, murine Kobuvirus 1.

Animals were exposed to microorganisms through three different models. SPF mice were housed in large enclosures with barn-type materials (soil and domestic animal excrements) (feralized, Fer), cohoused with wild mice in the previous condition (feralized-cohoused, Fer-CoH), or cohoused with pet store mice under barrier conditions (pet-cohoused, Pet-CoH; Fig. 1A). The first model represented microbial diversity from non-murine animal and environmental sources, while the latter two included microbes from different murine sources. After 78–84 days, animals were screened for pathogen exposure via serology (Fig. 1B). Fer-cohoused mice did not seroconvert for several natural mouse pathogens commonly found in the pet-cohoused system, including murine hepatitis virus, Sendai virus, pneumonia virus of mice, and *Mycoplasma pulmonis* (Fig. 1B). Surprisingly, murine astrovirus 2, the most common virus found in the pet store model (7), was also absent from the wild mice evaluated and no feralized-cohoused animals seroconverted (Fig. 1B). Conversely, all pet-cohoused mice seroconverted. To compare mice based on pathogen exposure across the models, a distance matrix was calculated using the serology panel results. We also compared these animals with 640 pet-cohoused mice previously published (7) and identified heterogenous pathogen transmission across each of the models (Fig. 1C). Feralized mice clustered closer to non-exposed SPF, reflecting the near lack of transmission of pathogens detected in the serology panel (Fig. 1B and C). Additionally, feralized-cohoused animals also clustered closer to SPF animals, suggesting that they have fewer canonical mouse pathogens than pet-cohoused mice (Fig. 1C). The viromes of wild mice used for feralizing-cohousing had not been previously evaluated. Using RNA sequencing, we only found reads for the picornaviruses murine Kobuvirus and Theiler’s murine encephalomyelitis virus (Fig. 1D). However, it is likely based on the serology data that these animals have been exposed to numerous other acute virus infections that may have cleared.

### The magnitude of vaccine responses is impacted by microbial experience

**Fig 2:**
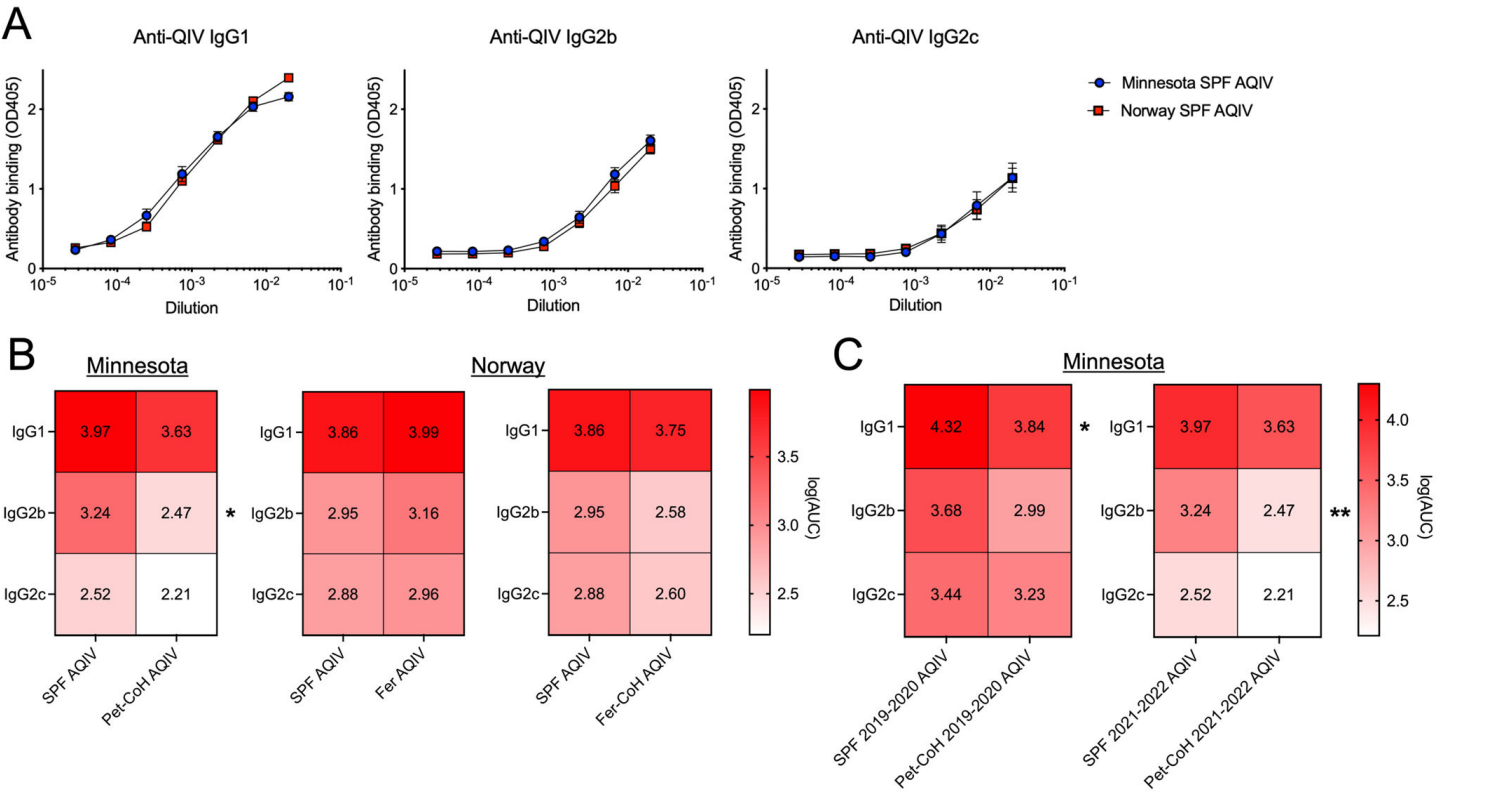
Seasonal influenza vaccine responses across model systems. SPF and microbe-exposed mice in Minnesota (cohoused for 98 days with pet store mice) and Norway (feralized for 98 days or feralized for 98 days and cohoused for 84 days with wild mice) were vaccinated with 2021–2022 seasonal quadrivalent influenza vaccine adjuvanted with Addavax, and vaccine-specific antibodies were measured 28–30 days post-vaccination by enzyme-linked immunosorbent assay. (A) Comparison of serum vaccine-specific IgG antibody levels in SPF mice between Minnesota and Norway. Antibody levels were compared using one-way analysis of variance (ANOVA). (B) Serum vaccine-specific IgG antibody response comparison between (left) SPF and pet-cohoused mice in Minnesota and (right) SPF, feralized, and feralized-cohoused mice in Norway. Antibody levels were determined by calculating log area under the curve (AUC) and compared using one-way ANOVA. (C) Serum vaccine-specific IgG antibodies in SPF and pet-cohoused mice vaccinated with either the 2019–2020 or 2021–2022 seasonal quadrivalent vaccines plus Addavax. Antibody levels were determined by calculating log(AUC) and compared using one-way ANOVA. 2019–2020 seasonal vaccination data from reference (7). *P < 0.05 and **P < 0.01.

To determine if the model of microbial experience impacts vaccination response, we inoculated pet-cohoused, feralized, feralized-cohoused, and SPF animals with the 2021–2022 seasonal quadrivalent influenza vaccine and measured the vaccine-specific antibodies 28–30 days post-vaccination. Importantly, there was no difference in the magnitude of vaccine-specific antibodies in SPF animals from Norway and Minnesota (Fig. 2A). In line with our previously published work, there was a diminished response to vaccination in pet-cohoused mice compared to SPF animals (Fig. 2B) (7). However, no defect in immunogenicity was observed in feralized or feralized-cohoused mice (Fig. 2B). Given the heterogeneity inherent to the pet store mouse cohousing model, we compared the vaccination across studies and different seasonal vaccine (2019–2020 quadrivalent influenza vaccine) strains. Using our previously published data we did not observe any differences in the magnitude of the response between 2019–2020 and 2021–2022 seasonal vaccines (Fig. 2C).

## DISCUSSION

The cohousing model is expanding in popularity (9, 11, 12). Differences in microbial experiences across these models offer an opportunity to evaluate the impact of heterogenous infections on the immune response. Microbial exposure can impact human immune responses to varying degrees. Both the microbiome and virome can influence the immune response to vaccination (13, 14). The microbiomes of people in low-income countries are more diverse than those in higher income countries. Some studies have linked these differences in microbiome diversity to disparities in vaccine efficacy (13). For example, the efficacy of the yellow fever vaccine 17D is reduced in Uganda compared to Switzerland, with people from Uganda having lower B and T cell responses (15). The virome also plays a role in immune responses to vaccination. In Ghana, detection of Cosavirus A and Enterovirus B is negatively associated with seroconversion after vaccination with a rotavirus vaccine (14). Together, these studies highlight the importance of microbial experience in immunity and how mouse models incorporating natural infections can help to better model the human condition. Further comparisons of mouse models incorporating microbial exposure will help to identify features that impact the immune response during infection, vaccinations, and other diseases.

The hygiene hypothesis predicts that microbial exposures will impact subsequent immune responses. The differential impact between commensals versus pathogens and acute versus chronic infections has not been well established, particularly in controlled laboratory settings. We found that different methods for introducing microbial experience into laboratory mice had varied impacts on a *de novo* immune response generated through vaccination. While the exposure and transmission windows were the same between the models, the degree of microbial experience was dramatically different. This is likely due to wild mice harboring fewer eukaryotic viruses than pet store mice (approximately two versus six eukaryotic viruses detected in the gastrointestinal (GI) tract and liver, respectively) (16, 17). Additionally, pet store mice transmit many acute viruses and parasites absent from wild mice in Norway, many of which are not captured by the SPF serology panel (Fig. 1) (16). Despite the lower number of microbes compared to both types of cohousing, feralized mice do experience an alteration in basal immune activation resulting in significant phenotypes (e.g., protection against colorectal cancer) (18). These data are consistent with a recent study which observed an impact of cohousing with both pet store and wild mice on the immune systems of SPF mice but was weaker with wild animals which may have fewer microbes (11).

While this study highlights potential differences across these model systems, there are several limitations. All three model systems drive changes in the microbiome, which have not been explored in this study and could be impacting vaccine responses. Baseline immunological assessments were performed differently due to study design limitations and are thus not directly comparable across models. Additionally, given the complexity of the model systems, each study was only performed once, though sufficient animals per group were included to have statistical power to evaluate differences in adaptive immune response. However, results from pet store cohousing are highly consistent with previous results generated using a different seasonal influenza vaccine (7). Altogether, utilizing a variety of models to introduce microbial experience to laboratory animals may be essential to understanding the heterogeneity of human immune responses to vaccination and infection.

## MATERIALS AND METHODS

### Models exposing mice to natural microbes and vaccination

For pet-cohousing (Minnesota), mice were acquired from local pet stores and cohoused with 8-week-old female C57BL/6 mice (The Jackson Laboratory) within a BSL-3 facility. For feralizing (Norway), female C57BL/6 mice were housed in large enclosures containing bedding, organic garden soil, and biweekly addition of fecal content from organically reared cows, sheep, poultry, and pigs, as previously described (18). For feralized-cohousing (Norway), mice were captured as previously described (19) and cohoused with female C57BL/6 (Janvier Labs, Saint-Berthevin Cedex, France) mice under feralizing conditions as described above.

C57BL/6 mice were either cohoused for 98 days with pet store mice, feralized for 98 days, or feralized for 98 days and cohoused for 84 days with wild mice. They were bled and screened for infectious agents using Multiplex Fluorometric ImmunoAssay Mouse Assessment Plus (Charles River Laboratories) or EZ-spot (IDEXX BioAnalytics). The Charles River panel detects *Encephalitozoon cuniculi* (ECUN), Ectromelia virus (ECTRO), mouse rotavirus (EDIM), *Filobacterium rodentium* (CARB), Theiler’s murine encephalitis virus (TMEV), hantavirus (HTNV), K virus, lymphocytic choriomeningitis virus (LCMV), lactate dehydrogenase elevating virus (LDV), murine adenoviruses (MAV1 and MAV2), murine cytomegalovirus (MCMV), murine hepatitis virus (MHV), murine norovirus (MNV), *Mycoplasma pulmonis* (MPUL), murine parvoviruses (MPV1, MPV2, NS-1), mouse thymic virus (MTLV), minute virus of mice (MVM), prospect hill virus (PHV), polyoma virus (POLY), pneumonia virus of mice (PVM), reovirus (REO), and Sendai virus (SEND). The IDEXX panel detects *Clostridium piliforme* (CPIL), ECUN, EDIM, TMEV, LECMV, MAV1 and MAV2, MCMV, MHV, MNV, MPUL, MPV, MVM, POLY, PVM, REO, SEND, and ECTRO. Age-matched control mice were housed in SPF facilities. Exposed and SPF mice were vaccinated intramuscularly with 1.8 µg (50 µL) of 2021–2022 seasonal influenza vaccine (Sanofi) diluted 1:1 with Addavax (InVivoGen). All experiments using mice were approved by the Institutional Animal Care and Use Committee at the University of Minnesota or the Norwegian Food Safety Authority (FOTS ID 27618) and Norwegian Environment Agency (2021/7274).

### Astrovirus serology

For murine astrovirus 2 detection, blood was collected and allowed to clot for 1 hour at room temperature before the serum was separated by spinning for 15 minutes at 3,000 rpm and then stored at −80°C. For murine astrovirus 2, the full length of the VP27 gene was constructed according to the contigs generated from the RNAseq data of pet store mice, as previously described (16). VP27 protein was expressed using baculovirus expression system, confirmed by SDS-PAGE and western blot analysis (Genscript). The recombinant VP27 protein was diluted in 0.1 M carbonate-bicarbonate buffer (pH 9.5) to a final concentration of 3 µg/mL and coated on 96-well plates (100 µL per well). Following adsorption of the antigen after incubation at 4°C overnight, the plates were washed with phosphate-buffered saline with Tween 20 (PBST) three times and then blocked with 5% skim milk at 37°C for 1.5 hours. Hundred microliters of mouse sera was incubated at a 1:40 dilution at 37°C for 1 hour, and subsequently washed three times with PBST. Bound antibody was detected as described below.

### Vaccine-specific antibody detection by enzyme-linked immunosorbent assay

After 28–30 days post-vaccination, blood was collected, and the serum was separated as described above. 96-well plates were coated with a 1:250 dilution of the 2021–2022 quadrivalent influenza vaccine (Sanofi Pasteur) diluted in phosphate buffered saline (PBS) and blocked with 1% bovine serum albumin (BSA) in PBS. Serial dilutions of mouse serum were added to the coated and blocked plates. Bound antibody was detected with horseradish peroxidase (HRP)-anti-mouse IgG1, IgG2b, and IgG2c antibodies (Southern Biotech) and detected by ABTS peroxidase substrate (SeraCare) on Synergy H1 plate reader (OD405) (BioTek).

### Virome evaluation

The liver, lung, small intestine, and large intestine of six wild mice were homogenized in GentleMacs M tubes (Miltenyi Biotec) in Buffer RLT Plus (Qiagen) supplemented with 2-mercaptoethanol (10 µL/1 mL) and Reagent DX (0.5% vol/vol; Qiagen). RNA was extracted using the AllPrep DNA/RNA mini kit (Qiagen). cDNA libraries of extracted RNA were prepared with Kapa Hyper prep kit (Roche) and sequenced with 150 bp paired-end reads on a NovaSeq machine. To identify eukaryotic viruses in sequencing reads, reads were mapped to the *Mus musculus* genome (release 109) with STAR (20). Unmapped reads were *de novo* assembled with Trinity (21), and taxonomy was identified using BLASTn (22). Read abundance was visualized in R using ggplot2 (23).

### Data analysis

To illustrate the similarities among cohoused mice based on past pathogen exposure, multiple correspondence analysis was performed with the FactoMineR package in R (24) using serology panel results (presence/absence of pathogens from EZ-spot and IDEXX mouse serology panels) from pet store-cohoused, feralized, wild, and feralized-cohoused mice.

Vaccine-specific antibody levels were determined by calculating the log area under the curve of each condition and compared using a one-way analysis of variance.

## Data Availability

Raw sequencing data are available under NCBI BioProject number PRJNA1062843 and the code is available at https://github.com/langloislab/sanders-et-al-2024.
